# Stability Analysis of SIR Model with Distributed Delay on Complex Networks

**DOI:** 10.1371/journal.pone.0158813

**Published:** 2016-08-04

**Authors:** Chuangxia Huang, Jie Cao, Fenghua Wen, Xiaoguang Yang

**Affiliations:** 1 School of Mathematics and Statistics, Changsha University of Science and Technology, Changsha, Hunan 410114, China; 2 Academy of Mathematics and Systems Science, Chinese Academy of Science, Beijing 10090, China; 3 School of Business, Central South University, Changsha, Hunan 410083, China; East China University of Science and Technology, CHINA

## Abstract

In this paper, by taking full consideration of distributed delay, demographics and contact heterogeneity of the individuals, we present a detailed analytical study of the Susceptible-Infected-Removed (SIR) epidemic model on complex population networks. The basic reproduction number $R_{0}$ of the model is dominated by the topology of the underlying network, the properties of individuals which include birth rate, death rate, removed rate and infected rate, and continuously distributed time delay. By constructing suitable Lyapunov functional and employing Kirchhoff’s matrix tree theorem, we investigate the globally asymptotical stability of the disease-free and endemic equilibrium points. Specifically, the system shows threshold behaviors: if $R_{0}\leq 1$, then the disease-free equilibrium is globally asymptotically stable, otherwise the endemic equilibrium is globally asymptotically stable. Furthermore, the obtained results show that SIR models with different types of delays have different converge time in the process of contagion: if $R_{0}>1$, then the system with distributed time delay stabilizes fastest; while $R_{0}\leq 1$, the system with distributed time delay converges most slowly. The validness and effectiveness of these results are demonstrated through numerical simulations.

## Introduction

The mathematical modeling of infectious disease propagation has been extensively studied by both medical practice and the academia for a long time (see, for instance, [1–7]). Such mathematical models focus on understanding the observed mechanisms of infectious diseases as well as predicting the consequences of the introduction of public health interventions to control the spreading of diseases [8]. In depicting the transmission of an infectious disease, the total population is commonly divided into susceptible, infectious and recovered individuals [9]. If the immunity is permanent, then we obtain an SIR model [7, 9]. The basic SIR model is formulated by Kermack and McKendrick [10] as the following:

$$\begin{array}{c} \left\{\begin{array}{c} \frac{dS(t)}{dt}=-\beta S\left(t\right)I\left(t\right),\\ \frac{dI(t)}{dt}=\beta S\left(t\right)I\left(t\right)-\lambda I\left(t\right),\\ \frac{dR(t)}{dt}=\lambda I\left(t\right), \end{array}\right. \end{array}$$ (1)

where *S*(*t*), *I*(*t*), *R*(*t*) represent the density of the susceptible, infected and removed (recovered) individuals, respectively; *β* is the transmission coefficient between susceptible and infected individuals, and λ is the recovery rate of infected individuals, the incidence at time *t* is denoted by *βS*(*t*)*I*(*t*). The SIR model (1) is built on the assumption that the individuals recover with immunity, which is appropriate for viral-agent-diseases such as measles, mumps, and smallpox. If the recovery does not give immunity, then the SIR model can be modified as the so called SIS model, which is applicable to diseases such as encephalitis and gonorrhea that caused by a bacterium. If individuals do not recover, then the SIS model can be changed into SI model [11]. For more details about these basic epidemiological models, we refer the interested reader to Refs. [7, 9, 11, 12]. In this paper, we mainly concentrate on the SIR epidemic model.

One of the key parameters is the basic reproduction number (sometimes called the basic reproductive ratio) $R_{0}$ in epidemiological investigations, which represents the expected number of secondary cases generated by one primary case of infection in a totally susceptible and sufficiently large population [13–15]. The basic reproduction number characterizes the transmission intensity of a particular disease in population and it is interpreted as a threshold criterion. For example, in model (1), if the initial number of susceptible individuals at the beginning of a disease is *S*_0_, then the basic reproduction number is $R_{0}=\beta S_{0}/\lambda$, if $R_{0}<1$, then the number of infected individuals declines monotonically, the epidemic eventually disappears; while $R_{0}>1$, the number of infected individuals increases first due to the infection and then decreases due to the recovery of infected individuals, there occurs outbreak and/or persistence of the disease [16].

Just as reported in [17], although model (1) provides an approximation for some observed disease data, the population in the system is under the assumption of both homogeneous infectivity and homogeneous connectivity of each individual, which is too oversimplified and ignores many structures of the real population. Different individuals may have varied number of acquaintances, and the transmission of many epidemic diseases exhibits heterogeneity [18, 19]. Nowadays, understanding the effects of the topological structures of population on the propagation of epidemics has attracted widespread attention [20]. One way to proceed would be to add contact heterogeneity to the population and see how much this alters the model behavior [17]. Many real world systems, such as social networks(stock financial network), biological systems (protein interactions), and technological systems (WWW, Internet) can be properly described as scale-free networks where nodes represent individuals or organizations and links mimic the interactions or connections among them [20–22]. A scale-free network is characterized by a power-law behavior *P*(*k*)∼*k*^−*γ*^, where *P*(*k*) is the probability that a node is connected to *k* other nodes and *γ* is a characteristic exponent whose value is usually in the range 2 < *γ* ≤ 3 [23–25]. Under this framework, each node of the network represents an individual in its corresponding state (susceptible, infected, or removed), possible contacts between two individuals are linked by an edge, and these two nodes are called neighbors of each other [17, 20]. Each edge is a connection along which the infection can spread, thus a node can acquire infection only from one of its neighbors, in other words, the contact rate is proportional to the number of neighbors, *i.e.* the degree of a node [17, 20].

Considering the contact heterogeneity in population, then model (1) can be generalized as below:

$$\begin{array}{c} \left\{\begin{array}{c} \frac{dS_{k}\left(t\right)}{dt}=-\beta kS_{k}\theta \left(t\right),\\ \frac{dI_{k}\left(t\right)}{dt}=\beta kS_{k}\theta \left(t\right)-\lambda I_{k}\left(t\right),\\ \frac{dR_{k}\left(t\right)}{dt}=I_{k}\left(t\right), \end{array}\right. \end{array}$$ (2)

where *S*_*k*_(*t*), *I*_*k*_(*t*) and *R*_*k*_(*t*) are the densities of susceptible, infected and recovered individuals with degree *k* (*k* = 1, 2, ⋯, *n*) at time *t*, respectively, *n* is the maximum degree of the network. *β* represents the transmission rate from susceptible individuals to infected individuals by contact to the infected individual in its neighbors. The term *θ*(*t*) denotes the probability that any given link points to an infected node [20]. Under the assumption of uncorrelated networks, the probability that a randomly chosen edge points to an infected node is $\theta \left(t\right)=\frac{\sum_{k}kP\left(k\right)I_{k}\left(t\right)}{\sum_{k}kP\left(k\right)}$, where *P*(*k*) is the degree distribution of the network [17, 20]. The term *βkS*_*k*_ *θ*(*t*) in system Eq (2) represents the incidence at the present time *t*. Model (2) offers a cognition of the propagation of epidemics, information and financial risk in complex systems. Since many real applications, such as infectious disease [19], information propagation [21], computer virus [26] and financial risks transmission [27] are all correlated with the epidemic dynamics on networks, more detailed justifications for epidemiology on networks have been carried out by some researchers, among which are J. Zhang *et al.* [3] and Y. Wang *et al.* [8], M. Small [18, 19], M. Newman [28], A. Cui *et al.* [29].

In traditional results, transmission on networks is dominated by the topology structure of the underlying network and the infection scheme, such as properties of disease, infection pattern, individual differences *etc.* [29–33]. In fact, during the propagation of epidemic, time delays do exist because an individual may not be infectious until some time after becoming infected [9, 34], some time (*τ*) is required before the infective organism develops in the vector to the level that is sufficient to pass the infection further [5–7]. When consider the influence of delay on disease spreading on networks, the models take a form of delay differential equation [25, 33, 35, 36]. In previous papers, time delay is assumed to be single-valued. As is well known, a constant delay may be applied if the movement time (*e.g.,* time until recovery or time until to be contagious) is known precisely, which is not very realistic for physical situation. For example, according to Anderson and May, measles virus has an infectious period of 5 − 7 days [15]. This variation may be due to variable environmental factors or the fact that healthy individuals recover faster. Allowing the infectivity to be varied in the interval since infection, up to some maximum duration, gives a model with a distributed delay [7, 9]. Therefore, it is more reasonable to introduce continuously distributed delays in epidemiology modeling. Unfortunately, to the best of the authors’ knowledge, few works have been done to study the transmission process that consider both the topological structures of the contact networks and distributed time delay. Analyzing the dynamics of the SIR model associated with distributed time delay on complex networks still remains a challenging open problem, which may deliver new insight of understanding the process of the propagation of the disease, information and financial risk, which motivates our study in this paper.

Generally speaking, in order to investigate the stability of time delayed SIR model, an efficient way is to use the Lyapunov’s second method, conditions in this method concern the long-time global dynamics. Of course, constructing suitable Lyapunov functional is usually not an easy task. By virtue of Kirchhoff’s matrix tree theorem, a novel Lyapunov functional is established in this paper. Our results make several contributions differing from the existing literature as follows. First of all, the disease-free equilibrium point is proven to be globally asymptotically stable when $R_{0}\leq 1$, and the endemic equilibrium point is globally asymptotically stable if $R_{0}>1$. Secondly, when the disease is permanence in the population, the densities of the infected individuals ascend if the degree of the node rises. What’s more, if *f* is larger, then the final density of the infected nodes will rise. Finally, we compare the propagation processes of disease with different types of delay through numerical simulations. By analyzing the final densities of infected individuals and the converge time of the propagation processes with distributed delay, discrete delay or without delay, we find that time delays accelerate the propagation of disease: when $R_{0}>1$, the system with distributed delay spreads fastest, the system without delay has slowest convergence rate, and the system without time delay performs between these two kinds of systems. Whereas $R_{0}\leq 1$, disease process under distributed delay lasts longest and the disease process without delay stabilizes fastest.

The remainder of this article is organized as follows. In section 2, based on some preliminaries and the assumptions, the model of epidemic associated with continuously distributed delay and complex networks are proposed. In section 3, by analyzing the corresponding characteristic equations and constructing suitable Lyapunov functional, the stability of disease-free equilibrium is studied. After then, by using Lyapunov functional and Kirchhoff’s matrix tree theorem, we investigate the stability of the endemic equilibrium. In Section 4, numerical examples are provided to demonstrate the validness and effectiveness of the obtained main results. The paper concludes with some remarks in Section 5.

## Epidemic model on networks

In order to address the effects of contact heterogeneity in epidemic spreading, the complex dispersal network G with *N* vertices is constructed as follows: each vertex represents an individual in its corresponding state (susceptible, infected, or removed), an undirected edge (*i*, *j*) is connected along which the infection can spread. We suppose that the adjacency matrix of the contact network G is irreducible, so that disease can disperse on the networks. Furthermore, in order to take account of the heterogeneity induced by the presence of nodes with different connectivities, we consider the time evolution of the magnitudes *S*_*k*_(*t*), *I*_*k*_(*t*) and *R*_*k*_(*t*), which denotes the densities of susceptible, infected and recovered individuals with degree *k* (*k* ∈ {1, 2, ⋯, *n*}, where *n* is the maximum degree of G) at time *t*, respectively, therefore, *S*_*k*_(*t*) + *I*_*k*_(*t*) + *R*_*k*_(*t*) = 1. To establish the propagation model on networks, throughout this paper, the following basic assumptions are imposed:

1. Suppose that the birth rate of the new individuals with degree *k* is *μ*, also, the death rate for each class is *μ*. The total number of the nodes with degree *k* in the network remains unchanged with time.
2. A healthy node can be infected at rate *β* if it is connected to infected individual.
3. The recovery rate of infected individuals is denoted by λ.
4. The probability that any randomly chosen edge points to an infected node at time *t* is denoted by *θ*(*t*). We assume that the contact network G is uncorrelated, then $\theta \left(t\right)=\frac{1}{\langle k\rangle }\sum_{k=1}^{n}kP(k)I_{k}\left(t\right)$.
5. Infectiousness varies over time, which is described by a integrable function *f*(*τ*) ≥ 0, *τ* ∈ [0, ∞) and $\int_{0}^{\infty }f\left(\tau \right)d\tau <+\infty$. The incidence at the present time *t* is $\beta kS_{k}\left(t\right)\int_{0}^{\infty }f\left(\tau \right)e^{-(\mu +\lambda )\tau }\theta \left(t-\tau \right)d\tau$, in which the term *e*^−(*μ* + λ)*τ*^ accounts for the probability of survival as infectious individual during latent period. According to [7, 9, 37], if $\int_{0}^{\infty }f\left(\tau \right)d\tau =1$, then the integrable function *f* is defined as the probability density function of transmission delay.

On the basis of the above assumptions, the population dynamics with degree *k* is written as follows

$$\begin{array}{c} \left\{\begin{array}{c} \frac{dS_{k}\left(t\right)}{dt}=-\beta kS_{k}\left(t\right)\int_{0}^{\infty }f\left(\tau \right)e^{-(\mu +\lambda )\tau }\theta \left(t-\tau \right)d\tau -\mu S_{k}\left(t\right)+\mu ,\\ \frac{dI_{k}\left(t\right)}{dt}=\beta kS_{k}\left(t\right)\int_{0}^{\infty }f\left(\tau \right)e^{-(\mu +\lambda )\tau }\theta \left(t-\tau \right)d\tau -\mu I_{k}\left(t\right)-\lambda I_{k}\left(t\right),\\ \frac{dR_{k}\left(t\right)}{dt}=\lambda I_{k}\left(t\right)-\mu R_{k}\left(t\right). \end{array}\right. \end{array}$$

Since the variable *R*_*k*_(*t*) does not appear in the equations of $\frac{dS_{k}\left(t\right)}{dt}$ and $\frac{dI_{k}\left(t\right)}{dt}$, furthermore, *R*_*k*_(*t*) = 1 − *I*_*k*_(*t*) − *S*_*k*_(*t*), which implies that it is sufficient to analyze the dynamic behaviours of

$$\begin{array}{c} \left\{\begin{array}{c} \frac{dS_{k}\left(t\right)}{dt}=-\beta kS_{k}\left(t\right)\int_{0}^{\infty }f\left(\tau \right)e^{-(\mu +\lambda )\tau }\theta \left(t-\tau \right)d\tau -\mu S_{k}\left(t\right)+\mu ,\\ \frac{dI_{k}\left(t\right)}{dt}=\beta kS_{k}\left(t\right)\int_{0}^{\infty }f\left(\tau \right)e^{-(\mu +\lambda )\tau }\theta \left(t-\tau \right)d\tau -\mu I_{k}\left(t\right)-\lambda I_{k}\left(t\right). \end{array}\right. \end{array}$$ (3)

The initial conditions for Eq (3) are represented as

$$S_{k}\left(0\right)\in R_{+0},I_{k}\left(u\right)=\phi_{k}\left(u\right),\phi_{k}\left(u\right)>0,u\in \left(-\infty ,0\right],$$ (4)

where *k* ∈ {1, 2, ⋯, *n*} is the degree of the node, $R_{+0}=\left[0,+\infty \right)$, $\phi_{k}\in C\left(\left(-\infty ,0\right],R_{+0}\right)$, $C(\left(-\infty ,0\right],R_{+0})$ is the positive cone of the Banach space of continuous functions mapping the interval (−∞, 0] into R equipped with the norm ‖*ϕ*‖ = sup_*u* ∈ (−∞, 0]_|*ϕ*(*u*)|.

**Remark 1.** We should point out that the assumptions (**i**), (**ii**), (**iii**), (**iv**), (**v**) are reasonable. Links between individuals would be cut off or new-established due to birth and death of individuals in population, so the demographics is considered in model (3). The propagation of disease on the network is described in an effective way: at each time step, a susceptible node is infected with probability *β*, if it is connected to one or more infected nodes, at the same time, an infected individual becomes removed with probability λ [20].

**Remark 2.** Although the time delayed epidemiology model on complex networks are studied in some papers [33, 36], the delays in all above-mentioned papers have been largely restricted to be discrete. In fact, for a biological reason, the epidemic propagation is not instantaneous and cannot be modeled with discrete delays, and a more appropriate way is to incorporate continuously distributed delays. Moreover, when the delay kernel *f* is chosen as a *δ* function at a certain time, then the distributed delay turns out to be discrete delay [38].

**Remark 3.** In this paper, we consider the heterogeneous contact network G. In fact, if the contact network is assumed to be homogeneous, such as Erdos-Reny network and WS small-world network, in which each individuals has same number of contacts, *i.e.*, *k* ≈ 〈*k*〉, then system Eq (3) degrades into

$$\begin{array}{c} \left\{\begin{array}{c} \frac{dS_{k}\left(t\right)}{dt}=-\beta \langle k\rangle S\left(t\right)\int_{0}^{\infty }e^{-(\mu +\lambda )\tau }f\left(\tau \right)I\left(t-\tau \right)d\tau -\mu S\left(t\right)+\mu ,\\ \frac{dI_{k}\left(t\right)}{dt}=\beta \langle k\rangle S\left(t\right)\int_{0}^{\infty }e^{-(\mu +\lambda )\tau }f\left(\tau \right)I\left(t-\tau \right)d\tau -\mu I\left(t\right)-\lambda I\left(t\right), \end{array}\right. \end{array}$$ (5)

where *S*(*t*), *I*(*t*) represents the density of susceptible individuals and the infected individuals, respectively. 〈*k*〉 is the number of contacts per unit time that is supposed to be constant for the whole population. Furthermore, if we treat *β*〈*k*〉 as a unitary coefficient, then the system Eq (5) can be dealt similarly as in [7, 9, 37].

## The stability of the equilibrium

### Spreading threshold and existence of the equilibriums

In order to find the equilibrium points of model (3), we denote

$$\begin{array}{c} R_{0}=\frac{\beta }{\mu +\lambda }\frac{\langle k^{2}\rangle }{\langle k\rangle }\hat{f}, \end{array}$$ (6)

where $\hat{f}=\int_{0}^{\infty }f\left(\tau \right)e^{-(\mu +\lambda )\tau }d\tau$, 〈*k*〉 and 〈*k*^2^〉 are the first and second moment of the degree respectively, *i.e.,* $\langle k\rangle =\sum_{k=1}^{n}kP\left(k\right)$, $\langle k^{2}\rangle =\sum_{k=1}^{n}k^{2}P\left(k\right)$. As usual, $\frac{\langle k^{2}\rangle }{\langle k\rangle }$ can be regarded as the index of heterogeneity of contact network G. We establish the following theorem.

**Theorem 1**. Consider the system Eq (3), we have the following assertions.

1. There always exists a disease-free equilibrium $E_{0}={\left(S_{1}^{0},S_{2}^{0},\ldots ;,S_{n}^{0},I_{1}^{0},I_{2}^{0},\ldots ;,I_{n}^{0}\right)}^{T}$, where $S_{k}^{0}=1$, $I_{k}^{0}=0$ for each *k* ∈ {1, 2, ⋯*n*}.
2. There is no endemic equilibrium if $R_{0}\leq 1$.
3. There is an unique endemic equilibrium $E_{+}={\left(S_{1}^{*},S_{2}^{*},\ldots ;,S_{n}^{*},I_{1}^{*},I_{2}^{*},\ldots ;,I_{n}^{*}\right)}^{T}$ if $R_{0}>1$, moreover,
  $$\begin{array}{c} S_{k}^{*}=\frac{\mu }{\beta k\hat{f}\theta^{*}+\mu },\;I_{k}^{*}=\frac{\mu \beta k\hat{f}\theta^{*}}{\left(\mu +\lambda \right)\left(\beta k\hat{f}\theta^{*}+\mu \right)},\;1\leq k\leq n, \end{array}$$ (7)
  where θ* is the unique positive root of the equation:
  $$\begin{array}{c} \frac{\mu }{\mu +\lambda }\sum_{k=1}^{n}a_{k}\frac{\beta k\hat{f}x}{\beta k\hat{f}x+\mu }=x\;\left(x\geq 0\right). \end{array}$$ (8)

**Proof.** It is obviously that *E*_0_ is always an equilibrium of systems Eq (3). Now let

$$\begin{array}{c} E_{+}=\left(S_{1}^{*},S_{2}^{*},\cdots ,S_{n}^{*},I_{1}^{*},I_{2}^{*},\cdots ,I_{n}^{*}\right) \end{array}$$

be the positive equilibrium of system Eq (3). For 1 ≤ *k* ≤ *n*, solve the equations

$$\begin{array}{c} \left\{\begin{array}{c} -\beta k\hat{f}S_{k}^{*}\theta^{*}-\mu S_{k}+\mu =0,\\ \beta k\hat{f}S_{k}^{*}\theta^{*}=\left(\mu +\lambda \right)I_{k}^{*}, \end{array}\right. \end{array}$$

then

$$\begin{array}{c} S_{k}^{*}=\frac{\mu }{\beta k\hat{f}\theta^{*}+\mu },I_{k}^{*}=\frac{\beta k\hat{f}\theta^{*}}{\mu +\lambda }S_{k}^{*}, \end{array}$$

therefore Eq (7) is obtained.

Since $\theta^{*}=\frac{1}{\langle k\rangle }\sum_{k=1}^{n}kP\left(k\right)I_{k}^{*}$, substituting $I_{k}^{*}$ into θ* yields

$$\begin{array}{c} \theta^{*}=\sum_{k=1}^{n}a_{k}\frac{\mu \beta k\hat{f}\theta^{*}}{\left(\mu +\lambda \right)\left(\beta k\hat{f}\theta^{*}+\mu \right)}. \end{array}$$ (9)

Because $I_{k}^{*}>0$, then θ* > 0 is a positive root of Eq (8).

Furthermore, define

$$\begin{array}{c} g\left(x\right)=\frac{\mu }{\mu +\lambda }\sum_{k=1}^{n}a_{k}\frac{\beta k\hat{f}x}{\beta k\hat{f}x+\mu }\;\left(x\geq 0\right), \end{array}$$ (10)

we have

$$\begin{array}{c} \left\{\begin{array}{c} g^{\prime }\left(x\right)=\frac{\mu }{\mu +\lambda }\sum_{k=1}^{n}a_{k}\frac{\mu \beta k\hat{f}}{{\left(\beta k\hat{f}x+\mu \right)}^{2}}{|}_{x\geq 0}>0,\\ g^{\prime \prime }\left(x\right)=\frac{\mu^{2}}{\mu +\lambda }\sum_{k=1}^{n}a_{k}\frac{-2\beta^{2}k^{2}{\hat{f}}^{2}}{{\left(\beta k\hat{f}x+\mu \right)}^{3}}{|}_{x\geq 0}<0,\\ g^{\prime }\left(0\right)=\frac{\mu }{\mu +\lambda }\sum_{k=1}^{n}a_{k}\frac{\beta k\hat{f}\mu }{\mu^{2}}=R_{0},\\ g(0)=0,\\ g\left(1\right)=\frac{\mu }{\mu +\lambda }\sum_{k=1}^{n}a_{k}\frac{\beta k\hat{f}}{\beta k\hat{f}+\mu }<1. \end{array}\right. \end{array}$$

If *g*′(0) ≤ 1, which implies $R_{0}\leq 1$, then *θ* = 0 is the single root of *g*(*x*) = *x*, thus the assertion (**ii**) of Theorem 1 holds. If *g*′(0)>1, which means $R_{0}>1$, function *g*(*x*) = *x* has a unique positive root, hence, the assertion (**iii**) of Theorem 1 holds as well, this completes the proof.

**Remark 4.** $R_{0}$ is called the basic reproduction number of system Eq (3). From Theorem 1, it is easy to see that $R_{0}$ exhibits threshold behaviors: if $R_{0}\leq 1$, the disease will be extinct gradually, otherwise, the disease will spread on networks. On one hand, according to [1, 20], $\frac{\langle k^{2}\rangle }{\langle k\rangle }$ is the parameter defining the level of heterogeneity of the network, then if the heterogeneity of network G rises, $R_{0}$ will be higher, the risk of disease outbreak becomes larger. Comparing to the Erdos-Renyi network and the small-world network, scale-free networks are very weak in face of infections [20]. On the other hand, $R_{0}$ gets larger if the function *f*(*τ*) is chosen larger in [0, ∞).

**Remark 5.** The unbounded distributed delay implies that the distant past has influence on the state of individual. What’s more, the unbounded distributed delay includes the bounded case by just choosing the function value equal to zero except for some bounded interval [38].

**Remark 6.** From Eq (7), it is obvious that $I_{k}^{*}=\frac{\mu \beta \hat{f}\theta^{*}}{\left(\mu +\lambda \right)\left(\beta \hat{f}\theta^{*}+\frac{\mu }{k}\right)}$, $I_{k}^{*}$ increases respect to *k*, *i.e.*, $I_{1}^{*}<I_{2}^{*}<\ldots ;I_{n}^{*}$, which implies the nodes with higher degree have higher infected densities densities eventually. Similarly, when *f* is chosen larger, then $I_{k}^{*}$ gets larger.

Let us define the set

$$\begin{array}{c} \Omega =\{\left(S_{1},S_{2},\cdots ,S_{n},I_{1},I_{2},\cdots ,I_{n}\right)\in R_{+}^{2n},0<S_{k}+I_{k}<1,1\leq k\leq n\}, \end{array}$$ (11)

where $R_{+0}^{2n}=\left\{\left(x_{1},x_{2},\ldots ;,x_{2n}\right)|x_{i}\geq 0\right\}$.

In the following, we will investigate positively invariant set for system Eq (3).

**Lemma 1.** The set Ω defined by Eq (11) is the positively invariant for system Eq (3).

**Proof.** From Eq (11), the set Ω can be rewritten as

$$\begin{array}{c} \Omega =\{X=\left(x_{1},x_{2},\cdots ,x_{2n}\right)|0<x_{l},\text{for}\;\;1\leq l\leq 2n;x_{i}+x_{i+n}\leq 1,\text{for}\;\;1\leq i\leq n\}, \end{array}$$ (12)

where *x*_*i*_ = *S*_*i*_ and *x*_*i*+*n*_ = *I*_*i*_ for 1 ≤ *i* ≤ *n*. The boundary of Ω, which is denoted by ∂Ω, consists of 3*n* flat sets

$$\begin{array}{ccc} & & \varrho_{i1}=\left\{X\in \Omega |x_{i}=0\right\},\\ & & \varrho_{i2}=\left\{X\in \Omega |x_{i+n}=0\right\},\\ & & \varrho_{i3}=\left\{X\in \Omega |x_{i}+x_{i+n}=1\right\},1\leq i\leq n, \end{array}$$

and the corresponding outer normal vectors are

$$\begin{array}{ccc} & & \xi_{i1}=\left(0,\cdots ,0,\overset{i}{-1},0,\cdots ,0\right),\\ & & \xi_{i2}=\left(0,\cdots ,0,\overset{i+n}{-1},0,\cdots ,0\right),\\ & & \xi_{i3}=\left(0,\cdots ,0,\overset{i}{1},0,\cdots ,0,\overset{i+n}{1},\cdots ,0\right),1\leq i\leq n. \end{array}$$

According to [39], Ω is positively invariant for system Eq (3), if for any point *X* in ∂Ω, $\frac{dX}{dt}$ is tangent or pointing into the set. For ∀*i* ∈ {1, 2, ⋯, *n*}, we have

$$\begin{array}{ccc} \left(\frac{dX}{dt}{|}_{x\in \varrho_{i1}},\xi_{i1}\right) & = & -1\cdot \left(-\beta iS_{i}\int_{0}^{\infty }f\left(\tau \right)e^{-(\mu +\lambda )\tau }\sum_{k=1}^{n}a_{k}I_{k}\left(t-\tau \right)d\tau -\mu S_{i}+\mu \right)\\ & = & -\mu \leq 0,\\ \left(\frac{dX}{dt}{|}_{x\in \varrho_{i2}},\xi_{i2}\right) & = & -(\beta iS_{i}\int_{0}^{\infty }f\left(\tau \right)e^{-(\mu +\lambda )\tau }\sum_{k=1}^{n}a_{k}I_{k}\left(t-\tau \right)d\tau )\leq 0,\\ \left(\frac{dX}{dt}{|}_{x\in \varrho_{i3}},\xi_{i3}\right) & = & \mu -\mu S_{i}-\mu I_{i}-\lambda I_{i}=-\lambda I_{i}\leq 0. \end{array}$$

Therefore, Ω is positively invariant for system Eq (3), this completes the proof.

### Stability of the disease-free equilibrium

**Theorem 2.** The disease-free equilibrium *E*_0_ of system Eq (3) is locally stable if $R_{0}<1$, and unstable if $R_{0}>1$.

**Proof.** Consider the disease-free equilibrium *E*_0_. Firstly, for each *k* ∈ {1, 2, ⋯, *n*}, set ${\tilde{S}}_{k}=S_{k}-1$, then the linearized system of system Eq (5) can be transformed into:

$$\left\{\begin{array}{c} \frac{d\tilde{S_{k}}}{dt}\left(t\right)=-\beta k\int_{0}^{\infty }f\left(\tau \right)e^{-(\mu +\lambda )\tau }\theta \left(t-\tau \right)d\tau -\mu {\tilde{S}}_{k},\\ \frac{dI_{k}\left(t\right)}{dt}=\beta k\int_{0}^{\infty }f\left(\tau \right)e^{-(\mu +\lambda )\tau }\theta \left(t-\tau \right)d\tau -\left(\mu +\lambda \right)I_{k}. \end{array}\right.$$ (13)

To study the stability of this linear system, we suppose the solutions for system Eq (13) with the exponential form as follows:

$$\begin{array}{c} {\tilde{S}}_{k}\left(t\right)={\tilde{S}}_{k0}e^{\rho t},\;I_{k}\left(t\right)=I_{k0}e^{\rho t},\left(k=1,2,\cdots ,n\right), \end{array}$$ (14)

where ${\tilde{S}}_{k0}$, *I*_*k*0_, *R*_*k*0_ represent the initial value. Substituting Eq (14) into Eq (13) yields

$$\begin{array}{c} \rho {\left({\tilde{S}}_{10},\cdots ,{\tilde{S}}_{n0},I_{10},\cdots ,I_{n0}\right)}^{T}=A{\left({\tilde{S}}_{10},\cdots ,{\tilde{S}}_{n0},I_{10},\cdots ,I_{n0}\right)}^{T}. \end{array}$$ (15)

Let $\Psi =\int_{0}^{\infty }f\left(\tau \right)e^{-(\rho +\mu +\lambda )\tau }d\tau$, where *ρ* is the eigenvalue of the matrix of system Eq (13), therefore,

$$\begin{array}{c} A={\left(\begin{array}{cccccc} -\mu & \cdots & 0 & -\beta a_{1}\Psi & \cdots & -\beta a_{n}\Psi \\ \vdots & \ddots & \vdots & \vdots & \ddots & \vdots \\ 0 & \cdots & -\mu & -\beta na_{1}\Psi & \cdots & -\beta na_{1}\Psi \\ 0 & \cdots & 0 & -\left(\mu +\lambda \right)+\beta a_{1}\Psi & \cdots & \beta a_{n}\Psi \\ \vdots & \ddots & \vdots & \vdots & \ddots & \vdots \\ 0 & \cdots & 0 & \beta na_{1}\Psi & \cdots & -\left(\mu +\lambda \right)+\beta na_{n}\Psi \end{array}\right)}_{2n\times 2n.} \end{array}$$

It is easy to see that the matrix *A* has *n* eigenvalues that equal to −*μ*. In order to obtain other eigenvalues of *A*, define

$$\begin{array}{c} F={\left(\begin{array}{cccc} -\left(\mu +\lambda \right)+\beta a_{1}\Psi & \beta a_{2}\Psi & \cdots & \beta a_{n}\Psi \\ \beta 2a_{1}\Psi & -\left(\mu +\lambda \right)+\beta 2a_{2}\Psi & \cdots & \beta 2a_{n}\Psi \\ \vdots & \vdots & \ddots & \vdots \\ \beta na_{1}\Psi & \beta na_{2}\Psi & \cdots & -\left(\mu +\lambda \right)+\beta na_{n}\Psi \end{array}\right)}_{n\times n.} \end{array}$$

Carry out similarity transformation to the matrix *F*: first of all, the second column multiplied by $-\frac{a_{1}}{a_{2}}$ is added to the first column, followed by analogy; secondly, the first row multiplied by $\frac{a_{1}}{a_{2}}$ is added to the second row, and followed by analogy, then we have

$$\begin{array}{c} F^{*}={\left(\begin{array}{cccc} -(\mu +\lambda ) & 0 & \cdots & \beta a_{n}\Psi \\ 0 & -(\mu +\lambda ) & \cdots & \beta 2a_{n}\Psi +\beta a_{n}\Psi \frac{a_{1}}{a_{2}}\\ \vdots & \vdots & \ddots & \vdots \\ 0 & 0 & \cdots & -\left(\mu +\lambda \right)+\beta \sum_{k=1}^{n}ka_{k}\Psi \end{array}\right)}_{n\times n}. \end{array}$$

It is obvious that the matrix F* has *n* − 1 eigenvalues that equal to −*μ* − λ, and the *n*th eigenvalue of F* is

$$\begin{array}{ccc} \Lambda & = & -\left(\mu +\lambda \right)+\beta \sum_{k=1}^{n}ka_{k}\int_{0}^{\infty }f\left(\tau \right)e^{-(\Lambda +\mu +\lambda )\tau }d\tau \\ & = & \left(\mu +\lambda \right) [\frac{\beta \langle k^{2}\rangle }{(\mu +\lambda )\langle k\rangle }\int_{0}^{\infty }f\left(\tau \right)e^{-(\Lambda +\mu +\lambda )\tau }d\tau -1]. \end{array}$$

Suppose that Λ = *a*+*bi*, then we have

$$\begin{array}{c} a+bi=\left(\mu +\lambda \right)R_{0}\frac{\int_{0}^{\infty }f\left(\tau \right)e^{-(a+\mu +\lambda )\tau }\left(\cos b\tau -i\sin b\tau \right)d\tau }{\hat{f}}-\left(\mu +\lambda \right) \end{array}$$

which implies that

$$\begin{array}{ccc} & & a=\left(\mu +\lambda \right) [R_{0}\frac{\int_{0}^{\infty }f\left(\tau \right)e^{-(a+\mu +\lambda )\tau }\cos b\tau d\tau }{\hat{f}}-1],\\ & & b=-\left(\mu +\lambda \right)R_{0}\frac{\int_{0}^{\infty }f\left(\tau \right)e^{-(a+\mu +\lambda )\tau }\sin b\tau d\tau }{\hat{f}}. \end{array}$$ (16)

Consider Eq (16), when $R_{0}<1$, if *a* ≥ 0, then the right side of Eq (16) is negative, which is a contradiction to *a* ≥ 0. Let $\varphi \left(x\right)=x-\left(\mu +\lambda \right)[R_{0}\frac{\int_{0}^{\infty }f\left(\tau \right)e^{-(x+\mu +\lambda )\tau }d\tau }{\hat{f}}-1]$, we have

$$\begin{array}{c} \frac{d\varphi (x)}{dx}=1+\left(\mu +\lambda \right)R_{0}\frac{\int_{0}^{\infty }f\left(\tau \right)\tau e^{-(x+\mu +\lambda )\tau }d\tau }{\hat{f}}>1, \end{array}$$

which means *φ*(*x*) is always monotonically increasing, what’s more

$$\begin{array}{c} \varphi \left(0\right)=-\left(\mu +\lambda \right)\left(R_{0}-1\right). \end{array}$$

If $R_{0}>1$, then *ϕ*(0)<0, the formula *ϕ*(*x*) = 0 has positive real root, the disease-free equilibrium *E*_0_ of system is unstable. If $R_{0}<1$, then *ϕ*(0)>0, therefore, all the root of *ϕ*(*x*) = 0 is negative, so the disease-free equilibrium *E*_0_ is locally asymptotically stable, this completes the proof.

**Theorem 3.** If $R_{0}\leq 1$, the disease-free equilibrium *E*_0_ is the unique equilibrium of system Eq (3), and it is globally asymptotically stable.

**Proof.** Choose the following Lyapunov function

$$V\left(t\right)=\sum_{k=1}^{n}a_{k}\int_{1}^{S_{k}\left(t\right)}\frac{x-1}{x}dx+\sum_{k=1}^{n}a_{k}I_{k}\left(t\right)+\frac{\mu +\lambda }{\hat{f}}\int_{0}^{\infty }f\left(\tau \right)e^{-(\mu +\lambda )\tau }\int_{t-\tau }^{t}\theta \left(v\right)dvd\tau ,$$ (17)

the time derivative of *V*(*t*) along the trajectories of system Eq (3) is given as

$$\begin{array}{ccc} \dot{V} & = & \sum_{k=1}^{n}a_{k}\frac{S_{k}\left(t\right)-1}{S_{k}\left(t\right)}\left(-\beta kS_{k}\left(t\right)\int_{0}^{\infty }f\left(\tau \right)e^{-(\mu +\lambda )\tau }\theta \left(t-\tau \right)d\tau -\mu S_{k}\left(t\right)+\mu \right)\\ & & +\sum_{k=1}^{n}a_{k}\left(\beta kS_{k}\left(t\right)\int_{0}^{\infty }f\left(\tau \right)e^{-(\mu +\lambda )\tau }\theta \left(t-\tau \right)d\tau -\left(\mu +\lambda \right)I_{k}\left(t\right)\right)\\ & & +\frac{\mu +\lambda }{\hat{f}}\int_{0}^{\infty }f\left(\tau \right)e^{-(\mu +\lambda )\tau }\theta \left(t\right)d\tau -\frac{\mu +\lambda }{\hat{f}}\int_{0}^{\infty }f\left(\tau \right)e^{-(\mu +\lambda )\tau }\theta \left(t-\tau \right)d\tau \\ & = & -\mu \sum_{k=1}^{n}a_{k}\frac{{\left(S_{k}\left(t\right)-1\right)}^{2}}{S_{k}\left(t\right)}+\sum_{k=1}^{n}a_{k}\beta k\int_{0}^{\infty }f\left(\tau \right)e^{-(\mu +\lambda )\tau }\theta \left(t-\tau \right)d\tau \\ & & -\sum_{k=1}^{n}a_{k}\left(\mu +\lambda \right)I_{k}\left(t\right)+\left(\mu +\lambda \right)\theta \left(t\right)-\frac{\mu +\lambda }{\hat{f}}\int_{0}^{\infty }f\left(\tau \right)e^{-(\mu +\lambda )\tau }\theta \left(t-\tau \right)d\tau . \end{array}$$

Since $\sum_{k=1}^{n}a_{k}I_{k}\left(\mu +\lambda \right)=\left(\mu +\lambda \right)\theta \left(t\right)$, then

$$\begin{array}{ccc} \dot{V} & = & -\mu \sum_{k=1}^{n}a_{k}\frac{{\left(S_{k}-1\right)}^{2}}{S_{k}}+\sum_{k=1}^{n}a_{k}\beta k\int_{0}^{\infty }f\left(\tau \right)e^{-(\mu +\lambda )\tau }\theta \left(t-\tau \right)d\tau \\ & & -\frac{\mu +\lambda }{\hat{f}}\int_{0}^{\infty }f\left(\tau \right)e^{-(\mu +\lambda )\tau }\theta \left(t-\tau \right)d\tau \\ & = & -\mu \sum_{k=1}^{n}a_{k}\frac{{\left(S_{k}\left(t\right)-1\right)}^{2}}{S_{k}\left(t\right)}+\frac{\mu +\lambda }{\hat{f}}\left(R_{0}-1\right)\int_{0}^{\infty }f\left(\tau \right)e^{-(\mu +\lambda )\tau }\theta \left(t-\tau \right)d\tau . \end{array}$$ (18)

Therefore, if $R_{0}\leq 1$, $\dot{V}\leq 0$. Furthermore, $\dot{V}=0$ only if *S*_*k*_(*t*) = 1, *I*_*k*_(*t*) = 0. Thus the system is globally asymptotically stable in Ω, this completes the proof.

### Stability of the endemic equilibrium

In order to investigate the stability of the endemic equilibrium, we shall demonstrate the Kirchhoff’s matrix tree theorem at first. Considering the matrix *Q* = (*q*_*ij*_)_*m*×*m*_, where *q*_*ij*_ ≥ 0, 1 ≤ *i*, *j* ≤ *m*, the directed graph *G*(*Q*) associated with *Q* = (*q*_*ij*_)_*m*×*m*_ has vertices {1, 2, ⋯, *m*} with a directed arc (*i*, *j*) from *i* to *j* iff *q*_*ij*_ ≠ 0. It is strongly connected if any two distinct vertices are joined by an oriented path. Matrix *Q* is irreducible if and only if *G*(*Q*) is strongly connected [40]. The Laplacian matrix of the directed graph *G*(*Q*) is defined as

$$\begin{array}{c} L=\left(\begin{array}{cccc} \sum_{l\neq 1}q_{1l} & -q_{21} & \cdots & -q_{m1}\\ -q_{12} & \sum_{l\neq 2}q_{2l} & \cdots & -q_{m2}\\ \vdots & \vdots & \ddots & \vdots \\ -q_{1m} & -q_{2m} & \cdots & \sum_{l\neq m}q_{ml} \end{array}\right). \end{array}$$

Let *C*_*ij*_ denote the cofactor of the (*i*, *j*) entry of *L*, then for the linear system

Lv=0, (19)

the following results holds.

**Lemma 2.** (Kirchhoff’s matrix tree theorem, see [41, 42]) Assume that *m* ≥ 2 and *Q* = (*q*_*ij*_)_*m*×*m*_ is irreducible, then following results hold:

1. The solution space of system Eq (19) has dimension 1, with a basis (*v*_1_, *v*_2_, ⋯, *v*_*m*_) = (*C*_11_, *C*_22_, ⋯, *C*_*mm*_).
2. For 1 ≤ *k* ≤ *m*,
  $$\begin{array}{c} C_{kk}=\sum_{T\in T_{k}}w\left(T\right)=\sum_{T\in T_{k}}\prod_{\left(r,l)\in E(T\right)}q_{rl}>0, \end{array}$$
  where $T_{k}$ is the set of all directed spanning subtrees of *G*(*Q*) that are rooted at vertex *k*, *w*(*T*) is the weight of a directed tree *T*, and *E*(*T*) denotes the set of directed arcs in *T*.

**Theorem 4.** If $R_{0}>1$, then the endemic equilibrium *E*_+_ is globally asymptotically stable in Ω∖{*E*_0_}.

**Proof.** When $R_{0}>1$, then from Theorem 1, we have

$$\begin{array}{c} \mu =\mu S_{k}^{*}+\beta k\hat{f}S_{k}^{*}\sum_{j=1}^{n}a_{j}I_{j}^{*},\;\left(\mu +\lambda \right)I_{k}^{*}=\beta k\hat{f}S_{k}^{*}\sum_{j=1}^{n}a_{j}I_{j}^{*}. \end{array}$$ (20)

Denoted by

$$V_{1k}\left(t\right)=S_{k}\left(t\right)-S_{k}^{*}-S_{k}^{*}\ln\frac{S_{k}\left(t\right)}{S_{k}^{*}},$$ (21)

$$V_{2k}\left(t\right)=I_{k}\left(t\right)-I_{k}^{*}-I_{k}^{*}\ln\frac{I_{k}\left(t\right)}{I_{k}^{*}},$$ (22)

$$V_{3k}\left(t\right)=\beta kS_{k}^{*}\sum_{j=1}^{n}a_{j}I_{j}^{*}\int_{0}^{\infty }\alpha \left(\tau \right)\psi \left(\frac{I_{j}\left(t-\tau \right)}{I_{j}^{*}}\right)d\tau ,j,k\in \left\{1,2,\cdots ,n\right\},$$ (23)

where

$$\psi \left(x\right)=x-1-\ln x,\;\text{and}\;\alpha \left(\tau \right)=\int_{\tau }^{\infty }f\left(x\right)e^{-(\mu +\lambda )x}dx.$$ (24)

We note that *V*_1*k*_(*t*) ≥ 0, *V*_2*k*_(*t*) ≥ 0 with equality if and only if $S_{k}\left(t\right)=S_{k}^{*},I_{k}\left(t\right)=I_{k}^{*}$. Also, *ψ*(*x*) has the strict global minimum *ψ*(1) = 0 for all *x* > 0, therefore *V*_3*k*_(*t*) ≥ 0 with equality if and only if $I_{j}\left(t-\tau \right)=I_{j}^{*}$ for all *τ* ∈ [0, *h*]. We will study the behavior of the Lyapunov functional as following

$$V_{k}\left(t\right)=V_{1k}\left(t\right)+V_{2k}\left(t\right)+V_{3k}\left(t\right).$$ (25)

The derivative of *V*_1*k*_(*t*) along system Eq (3) is given by

$$\begin{array}{ccc} & & \frac{dV_{1k}\left(t\right)}{dt}=\left(1-\frac{S_{k}^{*}}{S_{k}\left(t\right)}\right)\frac{dS(t)}{dt}\\ & = & -\mu \frac{{\left(S_{k}\left(t\right)-S_{k}^{*}\right)}^{2}}{S_{k}\left(t\right)}+\left(1-\frac{S_{k}^{*}}{S_{k}\left(t\right)}\right)\left(\beta k\hat{f}S_{k}^{*}\theta^{*}-\beta kS_{k}\left(t\right)\int_{0}^{\infty }f\left(\tau \right)e^{-(\mu +\lambda )\tau }\theta \left(t-\tau \right)d\tau \right). \end{array}$$

Similarly,

$$\begin{array}{c} \frac{dV_{2k}\left(t\right)}{dt}=\left(1-\frac{I_{k}^{*}}{I_{k}\left(t\right)}\right)\left(\beta kS_{k}\left(t\right)\int_{0}^{\infty }f\left(\tau \right)e^{-(\mu +\lambda )\tau }\theta \left(t-\tau \right)d\tau -\left(\mu +\lambda \right)I_{k}\left(t\right)\right). \end{array}$$

Therefore, we have

$$\begin{array}{ccc} & & \frac{dV_{1k}\left(t\right)}{dt}+\frac{dV_{2k}\left(t\right)}{dt}\\ & = & -\Delta_{k}+\beta kS_{k}^{*}\int_{0}^{\infty }f\left(\tau \right)e^{-(\mu +\lambda )\tau }\sum_{j=1}^{n}a_{j}I_{j}^{*}[2-\frac{S_{k}^{*}}{S_{k}\left(t\right)}+\frac{I_{j}\left(t-\tau \right)}{I_{j}^{*}}\\ & & -\frac{I_{k}\left(t\right)}{I_{k}^{*}}-\frac{S_{k}\left(t\right)I_{j}\left(t-\tau \right)I_{k}^{*}}{S_{k}^{*}I_{j}^{*}I_{k}\left(t\right)}]d\tau , \end{array}$$ (26)

where $\Delta_{k}=\mu \frac{{\left(S_{k}\left(t\right)-S_{k}^{*}\right)}^{2}}{S_{k}\left(t\right)}$.

Furthermore, the derivative of *V*_3*k*_(*t*) along Eq (3) is given by

$$\begin{array}{ccc} & & \frac{dV_{3k}\left(t\right)}{dt}=\frac{d}{dt}\beta kS_{k}^{*}\sum_{j=1}^{n}a_{j}I_{j}^{*}\int_{0}^{\infty }\alpha \left(\tau \right)\psi \left(\frac{I_{j}\left(t-\tau \right)}{I_{j}^{*}}\right)d\tau \\ & = & -\beta kS_{k}^{*}\sum_{j=1}^{n}a_{j}I_{j}^{*}\int_{0}^{\infty }\alpha \left(\tau \right)\frac{d}{d\tau }\psi \left(\frac{I_{j}\left(t-\tau \right)}{I_{j}^{*}}\right)d\tau . \end{array}$$

Using integration by parts, we obtain

$$\begin{array}{c} \frac{dV_{3k}\left(t\right)}{dt}=\beta kS_{k}^{*}\sum_{j=1}^{n}a_{j}I_{j}^{*} [-\alpha \left(\tau \right)\psi \left(\frac{I_{j}\left(t-\tau \right)}{I_{j}^{*}}\right){|}_{0}^{\infty }+\int_{0}^{\infty }\frac{d\alpha (\tau )}{d\tau }\psi \left(\frac{I_{j}\left(t-\tau \right)}{I_{j}^{*}}\right)d\tau ]. \end{array}$$ (27)

It follows from Eq (23) that $\lim_{h\to \infty }\alpha \left(h\right)=0$ and $\frac{d}{d\tau }\alpha \left(\tau \right)=-f\left(\tau \right)e^{-(\mu +\lambda )\tau }$, therefore,

$$\begin{array}{c} \frac{dV_{3k}\left(t\right)}{dt}=\beta kS_{k}^{*}\int_{0}^{\infty }\sum_{j=1}^{n}a_{j}I_{j}^{*}f\left(\tau \right)e^{-(\mu +\lambda )\tau } [\frac{I_{j}\left(t\right)}{I_{j}^{*}}-\frac{I_{j}\left(t-\tau \right)}{I_{j}^{*}}+\ln\frac{I_{j}\left(t-\tau \right)}{I_{j}\left(t\right)}]d\tau . \end{array}$$ (28)

From Eqs (26) and (28), we have

$$\begin{array}{ccc} \frac{dV_{k}\left(t\right)}{dt} & = & -\Delta_{k}+\beta kS_{k}^{*}\int_{0}^{\infty }f\left(\tau \right)e^{-(\mu +\lambda )\tau }\sum_{j=1}^{n}a_{j}I_{j}^{*}[2-\frac{S_{k}^{*}}{S_{k}\left(t\right)}-\frac{I_{k}\left(t\right)}{I^{*}}\\ & & -\frac{S_{k}\left(t\right)I_{j}\left(t-\tau \right)I_{k}^{*}}{S_{k}^{*}I_{j}^{*}I_{k}\left(t\right)}+\frac{I_{j}\left(t\right)}{I_{j}^{*}}+\ln\frac{I_{j}\left(t-\tau \right)}{I_{j}\left(t\right)}]d\tau . \end{array}$$ (29)

Since

$$\begin{array}{ccc} & & 2-\frac{S_{k}^{*}}{S_{k}\left(t\right)}-\frac{I_{k}\left(t\right)}{I^{*}}-\frac{S_{k}\left(t\right)I_{j}\left(t-\tau \right)I_{k}^{*}}{S_{k}^{*}I_{j}^{*}I_{k}\left(t\right)}+\frac{I_{j}\left(t\right)}{I_{j}^{*}}+\ln\frac{I_{j}\left(t-\tau \right)}{I_{j}\left(t\right)}\\ & = & \psi \left(\frac{I_{j}\left(t\right)}{I_{j}^{*}}\right)-\psi \left(\frac{I_{k}\left(t\right)}{I_{k}^{*}}\right)-\psi \left(\frac{S_{k}\left(t\right)I_{j}\left(t-\tau \right)I_{k}^{*}}{S_{k}^{*}I_{j}^{*}I_{k}\left(t\right)}\right)-\psi \left(\frac{S_{k}^{*}}{S_{k}\left(t\right)}\right)\\ & \leq & \psi \left(\frac{I_{j}\left(t\right)}{I_{j}^{*}}\right)-\psi \left(\frac{I_{k}\left(t\right)}{I_{k}^{*}}\right), \end{array}$$ (30)

denoted by $\psi \left(\frac{I_{j}\left(t\right)}{I_{j}^{*}}\right)=F_{j}$ and $\psi \left(\frac{I_{k}\left(t\right)}{I_{k}^{*}}\right)=F_{k}$, then

$$\begin{array}{ccc} \frac{dV_{k}\left(t\right)}{dt} & \leq & -\Delta_{k}+\beta kS_{k}^{*}\int_{0}^{\infty }f\left(\tau \right)e^{-(\mu +\lambda )\tau }\sum_{j=1}^{n}a_{j}I_{j}^{*}\left(F_{j}-F_{k}\right)d\tau \\ & = & -\Delta_{k}+\beta k\hat{f}S_{k}^{*}\sum_{j=1}^{n}a_{j}I_{j}^{*}\left(F_{j}-F_{k}\right). \end{array}$$ (31)

Consider the following two matrices

$$\begin{array}{ccc} B & = & {\left(\begin{array}{cccc} \beta \hat{f}a_{1}S_{1}I_{1} & \beta \hat{f}a_{2}S_{1}I_{2} & \cdots & \beta \hat{f}a_{n}S_{1}I_{n}\\ \beta \hat{f}2a_{1}S_{2}I_{1} & \beta \hat{f}2a_{2}S_{2}I_{2} & \cdots & \beta \hat{f}2a_{n}S_{2}I_{n}\\ \vdots & \vdots & \ddots & \vdots \\ \beta \hat{f}na_{1}S_{n}I_{1} & \beta \hat{f}na_{2}S_{n}I_{2} & \cdots & \beta \hat{f}na_{n}S_{n}I_{n} \end{array}\right)}_{n\times n},\\ \bar{B} & = & {\left(\begin{array}{cccc} \sum_{l\neq 1}{\bar{\beta }}_{1l} & -{\bar{\beta }}_{21} & \cdots & -{\bar{\beta }}_{n1}\\ -{\bar{\beta }}_{12} & \sum_{l\neq 2}{\bar{\beta }}_{2l} & \cdots & -{\bar{\beta }}_{n2}\\ \vdots & \vdots & \ddots & \vdots \\ -{\bar{\beta }}_{1n} & -{\bar{\beta }}_{2n} & \cdots & \sum_{l\neq n}{\bar{\beta }}_{nl} \end{array}\right)}_{n\times n}, \end{array}$$

and set ${\bar{\beta }}_{kj}=\beta k\hat{f}a_{j}S_{k}^{*}I_{j}^{*},1\leq j,k\leq n$. Since ${\bar{\beta }}_{kj}>0$, the digraph *G*(*B*) associated with *B* is strongly connected, then matrix *B* is irreducible, thus the Laplacian matrix $\bar{B}$ of *G*(*B*) is irreducible. Let *C*_*kj*_ denote the cofactor of the (*k*, *j*) entry of $\bar{B}$, from Lemma 2, we know that system $\bar{B}c=0$ has a positive solution *c* = (*c*_1_, *c*_2_, ⋯, *c*_*n*_), where *c*_*k*_ = *C*_*kk*_ > 0 for any *k* = 1, 2, ⋯, *n*.

Choose the Lyapunov functional as $V\left(t\right)=\sum_{k=1}^{n}c_{k}V_{k}\left(t\right),$ from Eq (31), we have

$$\begin{array}{ccc} \frac{dV(t)}{dt} & \leq & \sum_{k=1}^{n}c_{k} [-\Delta_{k}+\beta k\hat{f}S_{k}^{*}\sum_{j=1}^{n}a_{j}I_{j}^{*}\left(F_{j}-F_{k}\right)]\\ & = & -\sum_{k=1}^{n}c_{k}\Delta_{k}+\sum_{k,j=1}^{n}c_{k}{\bar{\beta }}_{kj}\left(F_{j}-F_{k}\right)=-\sum_{k=1}^{n}c_{k}\Delta_{k}+H. \end{array}$$ (32)

Next, we will show that $H=\sum_{k,j=1}^{n}c_{k}{\bar{\beta }}_{kj}\left(F_{j}-F_{k}\right)=0$. In fact, since *Bc* = 0, we have $\sum_{j=1}^{n}{\bar{\beta }}_{jk}c_{j}=\sum_{i=1}^{n}{\bar{\beta }}_{ki}c_{k}$, therefore, $\sum_{k,j=1}^{n}c_{k}{\bar{\beta }}_{kj}F_{j}=\sum_{k=1}^{n}F_{k}\sum_{j=1}^{n}{\bar{\beta }}_{kj}c_{k}=\sum_{k,j=1}^{n}c_{k}{\bar{\beta }}_{kj}F_{k},$ which implies that *H* ≡ 0 for all *I*_1_(*t*), *I*_2_(*t*), ⋯, *I*_*n*_(*t*). Then $\frac{dV(t)}{dt}\leq 0$ holds for all $\left(S_{1}^{*},S_{2}^{*},\ldots ;,S_{n}^{*},I_{1}^{*},I_{2}^{*},\ldots ;,I_{n}^{*}\right)$.

Furthermore, since Δ_*k*_ = 0, then $S_{k}\left(t\right)=S_{k}^{*}$, moreover, $\psi \left(\frac{I_{k}\left(t\right)}{I_{k}^{*}}\right)-\psi \left(\frac{I_{j}\left(t\right)}{I_{j}^{*}}\right)=0$ when $\frac{I_{k}\left(t\right)}{I_{k}^{*}}=\frac{I_{j}\left(t\right)}{I_{j}^{*}}=\sigma$. Therefore, $\frac{dV(t)}{dt}=0$ if and only if

$$S_{k}\left(t\right)=S_{k}^{*},\;\text{and}\;I_{k}\left(t\right)=\sigma I_{k}^{*},k\in \left\{1,2,\cdots ,n\right\},t>0,\sigma >0.$$ (33)

Substituting Eq (33) into the first equation of system Eq (3), we have

$$0=\mu -\mu S_{k}^{*}-\sigma \beta k\hat{f}S_{k}^{*}\theta^{*}.$$ (34)

From Eq (7), we know that Eq (34) holds when *σ* = 1, namely at *E*_+_. Therefore, the only compact invariant subset of the set where $\frac{dV(t)}{dt}=0$ is {*E*_+_}, thus the endemic equilibrium *E*_+_ is globally asymptotically stable.

**Remark 7.** By using Lyapunov functional and Kirchhoff’s matrix tree theorem [41, 42], the global stability of the endemic points is proved. We note that the Kirchhoff’s matrix tree theorem has been already employed in some previous papers, such as [40–43], unfortunately, these articles mainly concentrate on the time delay, and the influences of contact heterogeneity of the system are ignored. In this paper, the dynamical behaviors of the propagation model that influenced by both distributed time delay and heterogeneity topology are investigated. Therefore, our results complement the relative investigations in [40–43].

**Remark 8.** The results in this paper show clearly that the basic reproduction number $R_{0}$ of the contagion model not only depends on the epidemic properties, but also depends on the topology structure of the population networks and time delay. According to Eq (6), it is obvious that $R_{0}$ is proportional to the connectivity fluctuations 〈*k*^2^〉 and infected rate. Therefore, for the sake of preventing the disease from breaking out, in practice, taking quarantine measures contrary to some specific populations (such as hub nodes and so on) to reduce the heterogeneity of the network is an efficient option.

## Numerical examples

In this section, three examples are presented to demonstrate the correctness and effectiveness of the main obtained results in this paper.

We start with a graph with 5 vertexes, in each time step, a node with one edge is added into the graph and by using preferential attachment mechanism in [24], a BA scale-free network $G_{1}$ with 800 nodes is obtained (see S1 Fig). The following simulations are implemented based on $G_{1}$. The minimal degree in $G_{1}$ is 1 and the maximum degree is 70, the corresponding degree distribution is shown in S2 Fig. By simply computing, the first moment of degree is 〈*k*〉 = 3.9733 and the second moment of degree is 〈*k*^2^〉 = 44.8200.

**Example 1** Based on $G_{1}$, for *k* ∈ {1, 2, ⋯, 70}, consider the following SIR epidemic model

$$\begin{array}{c} \left\{\begin{array}{c} \frac{dS_{k}\left(t\right)}{dt}=-0.06kS_{k}\left(t\right)\int_{0}^{\infty }0.5e^{-1.3\tau }\theta \left(t-\tau \right)d\tau -0.4S_{k}\left(t\right)+0.4,\\ \frac{dI_{k}\left(t\right)}{dt}=0.06kS_{k}\left(t\right)\int_{0}^{\infty }0.5e^{-1.3\tau }\theta \left(t-\tau \right)d\tau -0.4I_{k}\left(t\right)-0.4I_{k}\left(t\right), \end{array}\right. \end{array}$$ (35)

with the initial conditions

$$\begin{array}{c} S_{k}\left(0\right)=\frac{0.7kP(k)}{44.8200},I_{k}\left(u\right)=\frac{0.3kP(k)}{44.8200},u\in \left(-\infty ,0\right]. \end{array}$$

It is obvious that *β* = 0.06, λ = 0.4, *μ* = 0.4, *f*(*τ*) = 0.5*e*^−0.5*τ*^, 0 ≤ *τ* ≤ ∞ and the assumptions (**i**), (**ii**), (**iii**), (**iv**), (**v**) are satisfied.

To show the dynamical behaviors of propagation process, we perform Monte Carlo simulations in this paper. In a time step, every infected individual tries to infect each of its susceptible neighbors with infection rate 0.06, and it cures itself with rate 0.4, the time delay is distributed from 0 to ∞. By simply computing, one can get that the positive invariant set of system Eq (35) is $\bar{\Omega }=\left\{0\leq S_{k}\left(t\right),0\leq I_{k}\left(t\right),0\leq S_{k}\left(t\right)+I_{k}\left(t\right)\leq 1,1\leq k\leq 70\right\}$ and the basic reproduction number is $R_{0}=\frac{\beta }{\mu +\lambda }\frac{\langle k^{2}\rangle }{\langle k\rangle }\int_{0}^{\infty }f\left(\tau \right)e^{-(\mu +\lambda )\tau }d\tau =0.7025<1$, thus the conditions for Theorem 3 are all satisfied. Furthermore, by using Monte Carlo simulations, the dynamical behaviors of susceptible nodes and the infected nodes are presented in S3 and S4 Figs. Two properties of the propagation process are shown in S3 Fig: for each *k* ∈ {1, 2, ⋯70}, the time series and the densities of susceptible nodes *S*_*k*_(*t*) are presented in S3 Fig, and *S*_*k*_(*t*) converges to 1 eventually; similarly in S4 Fig, the densities of infected nodes *I*_*k*_(*t*) are shown, which converges to zero gradually. The simulations in S3 Fig imply that the orbit of the system Eq (35) converges to the disease-free equilibrium *E*_0_ = (*S*_1_, *S*_2_, ⋯, *S*_70_, *I*_1_, *I*_2_, ⋯, *I*_70_), where *S*_*k*_ = 1, *I*_*k*_ = 0, 1 ≤ *k* ≤ 70, therefore, *E*_0_ is globally asymptotically stable in region $\bar{\Omega }$, which agrees with Theorem 3.

**Example 2** For *k* ∈ {1, 2, ⋯, 70}, consider the following SIR epidemic model on $G_{1}$

$$\begin{array}{c} \left\{\begin{array}{c} \frac{dS_{k}\left(t\right)}{dt}=-0.2kS_{k}\left(t\right)\int_{0}^{\infty }0.5e^{-1.2\tau }\theta \left(t-\tau \right)d\tau -0.4S_{k}\left(t\right)+0.4,\\ \frac{dI_{k}\left(t\right)}{dt}=0.2kS_{k}\left(t\right)\int_{0}^{\infty }0.5e^{-1.2\tau }\theta \left(t-\tau \right)d\tau -0.4I_{k}\left(t\right)-0.3I_{k}\left(t\right), \end{array}\right. \end{array}$$ (36)

with the initial function

$$\begin{array}{c} S_{k}\left(0\right)=\frac{0.7kP(k)}{44.8200},I_{k}\left(u\right)=\frac{0.3kP(k)}{44.8200},u\in \left(-\infty ,0\right], \end{array}$$

where *β* = 0.2, λ = 0.3, *μ* = 0.4, *f*(*τ*) = 0.5*e*^−0.5*τ*^, 0 ≤ *τ* ≤ ∞, the initial condition Eq (4) and the assumptions (**i**), (**ii**), (**iii**), (**iv**), (**v**) are satisfied.

We present Monte Carlo simulations of the propagation process to show the validness of the results in this paper. In each step, a susceptible can become infected at the rate of 0.2 and an infected node would become recovery at the rate of 0.3, the time delay is distributed from 0 to ∞. By simply computing, one can obtain that $R_{0}=\frac{\beta }{\mu +\lambda }\frac{\langle k^{2}\rangle }{\langle k\rangle }\int_{0}^{\infty }f\left(\tau \right)e^{-(\mu +\lambda )\tau }d\tau =1.5680>1$, and the invariant set of system Eq (35) is ${\bar{\Omega }}_{1}=\left\{0\leq S_{k}\left(t\right),0\leq I_{k}\left(t\right),0\leq S_{k}\left(t\right)+I_{k}\left(t\right)\leq 1,1\leq k\leq 70\right\}\backslash {\bar{E}}_{0}$, where ${\bar{E}}_{0}=\left(S_{1},S_{2},\ldots ;,S_{70},I_{1},I_{2},\ldots ;,I_{70}\right)$, and *S*_*k*_ = 1, *I*_*k*_ = 0, 1 ≤ *k* ≤ 70, thus all the conditions for Theorem 4 are satisfied. With the help of Monte Carlo simulations, the dynamical behaviors of susceptible nodes and infected nodes are shown in S5 and S6 Figs, which depict the propagation process clearly. In S5 Fig, the time series and the densities of susceptible nodes (*S*_*k*_(*t*), *k* ∈ {1, 2, ⋯70}) are shown, for each *k* ∈ {1, 2, ⋯70}, *S*_*k*_(*t*) converges to $S_{k}^{*}=\frac{0.4}{0.0197k+0.4}$ eventually; while in S6 Fig, the densities of infected nodes *I*_*k*_(*t*) are presented, which converge to positive steady levels $I_{k}^{*}=\frac{0.079k}{0.1378k+2.8}$. S5 and S6 Figs show that the orbits of system Eq (36) converge to the endemic equilibrium $E_{+}=\left\{S_{1}^{*},S_{2}^{*},\ldots ;,S_{70}^{*},I_{1}^{*},I_{2}^{*},\ldots ;,I_{k}^{*}\right\}$ eventually, therefore, *E*_+_ is globally asymptotically stable in region ${\bar{\Omega }}_{1}$, which agrees with Theorem 4. Furthermore, S6 Fig shows that those nodes with higher degree have higher infected densities, which is consistent with Remark 5.

**Example 3** In order to figure out the influences of delay, basing on $G_{1}$, several cases are arranged here.

**Case 1.** Consider the SIR model (3) with a distributed delay:

$$\begin{array}{c} \left\{\begin{array}{c} \frac{dS_{k}\left(t\right)}{dt}=-0.06kS_{k}\left(t\right)\int_{0}^{\infty }0.5e^{-1.45\tau }\theta \left(t-\tau \right)d\tau -0.5S_{k}\left(t\right)+0.5,\\ \frac{dI_{k}\left(t\right)}{dt}=0.06kS_{k}\left(t\right)\int_{0}^{\infty }0.5e^{-1.45\tau }\theta \left(t-\tau \right)d\tau -0.45I_{k}\left(t\right)-0.5I_{k}\left(t\right), \end{array}\right. \end{array}$$ (37)

and the initial conditions are supposed as the following

$$\begin{array}{c} S_{k}\left(0\right)=\frac{0.7kP(k)}{44.8200},I_{k}\left(u\right)=\frac{0.3kP(k)}{44.8200},u\in \left(-\infty ,0\right],k\in \left\{1,2,\cdots ,70\right\}, \end{array}$$ (38)

*i.e*, *β* = 0.06, λ = 0.45, *μ* = 0.5, and *f*(*τ*) = 0.5*e*^−0.5*τ*^. To show the differences of delays clearly, we consider the total density of the infected nodes *I*(*t*), which is expressed by an average over the various connectivity classes, *i.e.*, $I\left(t\right)=\sum_{k=1}^{70}P\left(k\right)I_{k}\left(t\right)$ [20]. From Eq (6), the basic reproductive number is $R_{0}=0.7001$. The total density *I*(*t*) converges to 0, which agrees with Theorem 3, *i.e.*, the disease-free equilibrium of system Eq (37) is globally asymptotically stable. The trajectory of *I*(*t*) of system Eq (37) when $R_{0}=0.8532$ is shown in S5 Fig.

**Case 2.** Consider the following SIR model with a discrete delay

$$\begin{array}{c} \left\{\begin{array}{c} \frac{dS_{k}\left(t\right)}{dt}=-0.06kS_{k}\left(t\right)\theta \left(t-h\right)-0.5S_{k}\left(t\right)+0.5,\\ \frac{dI_{k}\left(t\right)}{dt}=0.06kS_{k}\left(t\right)\theta \left(t-h\right)-0.45I_{k}\left(t\right)-0.5I_{k}\left(t\right), \end{array}\right. \end{array}$$ (39)

with initial conditions Eq (38), and the discrete delay is chosen as *h* = 300. According to [35, 36], one can obtain that the reproduction number of Eq (39) is $R_{0}=0.5568$. The total density *I*(*t*) converges to 0, which implies that the disease-free equilibrium of the system is globally asymptotically stable. The corresponding trajectory of *I*(*t*) of systems Eq (39) is shown in S7 Fig.

**Case 3.** Consider the following SIR model without time delay, *i.e.*, an SIR model in an ODE type:

$$\begin{array}{c} \left\{\begin{array}{c} \frac{dS_{k}\left(t\right)}{dt}=-0.06kS_{k}\left(t\right)\theta \left(t\right)-0.5S_{k}\left(t\right)+0.5,\\ \frac{dI_{k}\left(t\right)}{dt}=0.06kS_{k}\left(t\right)\theta \left(t\right)-0.45I_{k}\left(t\right)-0.5I_{k}\left(t\right), \end{array}\right. \end{array}$$ (40)

with identical initial conditions Eq (38). According to [3], the reproduction number of Eq (40) is $R_{0}=0.5568$. *I*(*t*) converges to 0 eventually. When $R_{0}=0.6839$, the corresponding trajectory of *I*(*t*) of system Eq (40) is shown in S5 Fig.

All the system trajectories in S5 Fig show that the total densities of the infected nodes converge to zero eventually when $R_{0}\leq 1$. More specially, the system Eq (40) without delay converges fastest, the system Eq (37) with distributed time delay stabilizes the most slowly, and the system Eq (39) with discrete delay performs between systems Eqs (40) and (37). In fact, by simple computation, one can obtain that the convergence time of system Eq (37) is 18.6200, while the convergence time of system Eq (39) is 11.8800 and for system Eq (40), the convergence time is 9.3100 (if ‖*I*(*t*)‖^2^ ≤ 0.0001 for all *t* ≥ t*, then we call t* as the convergence time). The convergence time of the SIR systems are listed in Table 1.

**Table 1:** System, convergence time, and the final density of infected individuals when $R_{0}<1$.

System	Type of delay	$R_{0}$	Convergence time of system	Final density of infected individuals
system Eq (37)	distributed delay	0.8532	18.6200	0
system Eq (39)	discrete delay	0.5568	11.8800	0
system Eq (40)	without delay	0.5568	9.3100	0

Furthermore, when $R_{0}>1$, let us consider the following cases.

**Case 4.** Consider the following SIR model with a distributed delay:

$$\begin{array}{c} \left\{\begin{array}{c} \frac{dS_{k}\left(t\right)}{dt}=-0.5kS_{k}\left(t\right)\int_{0}^{\infty }0.5e^{-1.1\tau }\theta \left(t-\tau \right)d\tau -0.3S_{k}\left(t\right)+0.3,\\ \frac{dI_{k}\left(t\right)}{dt}=0.5kS_{k}\left(t\right)\int_{0}^{\infty }0.5e^{-1.1\tau }\theta \left(t-\tau \right)d\tau -0.3I_{k}\left(t\right)-0.3I_{k}\left(t\right), \end{array}\right. \end{array}$$ (41)

with the initial conditions Eq (38), therefore, *β* = 0.5, λ = 0.3, *μ* = 0.3, and *f*(*τ*) = 0.5*e*^−0.5*τ*^. By using Eq (6), the basic reproductive number $R_{0}=10.6779$. The total density *I*(*t*) converges to I* = 0.3450, which agrees with Theorem 4 and implies that the endemic equilibrium of system Eq (41) is globally stable. The trajectory of *I*(*t*) of system Eq (41) is shown in S6 Fig.

**Case 5.** Consider the following SIR model with a discrete delay

$$\begin{array}{c} \left\{\begin{array}{c} \frac{dS_{k}\left(t\right)}{dt}=-0.5kS_{k}\left(t\right)\theta \left(t-h\right)-0.3S_{k}\left(t\right)+0.3,\\ \frac{dI_{k}\left(t\right)}{dt}=0.5kS_{k}\left(t\right)\theta \left(t-h\right)-0.3I_{k}\left(t\right)-0.3I_{k}\left(t\right), \end{array}\right. \end{array}$$ (42)

with initial conditions Eq (38), similarly, we choose *h* = 300. According to [35, 36], the reproduction number of Eq (42) is $R_{0}=8.5720$. *I*(*t*) converges to I* = 0.3155 eventually, therefore, the endemic equilibrium of system Eq (42) globally asymptotically stable, the corresponding trajectory is shown in S6 Fig.

**Case 6.** Consider the following SIR model without time delay, *i.e.*, an SIR model in an ODE type:

$$\begin{array}{c} \left\{\begin{array}{c} \frac{dS_{k}\left(t\right)}{dt}=-0.5kS_{k}\left(t\right)\theta \left(t\right)-0.3S_{k}\left(t\right)+0.3,\\ \frac{dI_{k}\left(t\right)}{dt}=0.5kS_{k}\left(t\right)\theta \left(t\right)-0.3I_{k}\left(t\right)-0.3I_{k}\left(t\right), \end{array}\right. \end{array}$$ (43)

the initial conditions are chosen as Eq (38). According to [3], the reproduction number of Eq (40) is $R_{0}=8.5720$. The total density *I*(*t*) converges to I* = 0.3155, which means that the endemic equilibrium of system Eq (43) is stable. The corresponding trajectory of *I*(*t*) of system Eq (43) is shown in S8 Fig.

Similarly, we define ${\tilde{t}}^{*}$ as the convergence time, if ‖*I*(*t*) − I*‖^2^ ≤ 0.0001 for all $t\geq {\tilde{t}}^{*}$, where I* is the final density of the infected individuals. The convergence time of the SIR models and the final densities of systems Eqs (41)–(43) are listed in Table 2. From Table 2, one can see that the convergence time of system Eq (41) is 4.0100, the convergence time of system Eq (42) is 8.9400, and for system Eq (43), the convergence time is 9.6100. The system Eq (41) with distributed time stabilizes fastest, and the system Eq (43) without time delay converges the most slowly.

**Table 2:** System, the convergence time and the final density of infected individuals when $R_{0}>1$.

System	Type of delay	$R_{0}$	Convergence time of system	Final density of infected individuals
system Eq (41)	distributed delay	10.6779	4.0100	0.3450
system Eq (42)	discrete delay	8.5720	8.9400	0.3155
system Eq (43)	without delay	8.5720	9.6100	0.3155

From above results, it is obvious that time delay has significant effects on the spreading of epidemic. As is indicated in S5 and S6 Figs, when $R_{0}\leq 1$, time delays slow down the extinction of disease, while $R_{0}>1$, time delays accelerate the spreading of disease. More specifically, by comparing the convergence time of each system, the results show that the differences of delays influence the propagation: when the distributed delay is incorporated, if $R_{0}\leq 1$, then the disease lasts longest, while $R_{0}>1$, then the disease spreads fastest.

## Conclusion

In this paper, by considering distributed delay, demographics and contact heterogeneity of the individuals, we study SIR model on complex population networks in detail. We prove that there exists the basic reproduction number of the epidemic $R_{0}$ which determines not only the existence of the endemic equilibrium *E*_+_ but also the stability of *E*_+_ and the disease-free equilibrium *E*_0_. Specifically, the epidemic will be extinct gradually if $R_{0}\leq 1$; while if $R_{0}>1$, the disease becomes permanence and the endemic equilibrium point is globally asymptotically stable. A series of numerical experiments are presented to confirm the correctness of the theoretical analysis as well. Furthermore, the numerical simulations show that the distributed delay slows down the extinction of disease when $R_{0}\leq 1$; while $R_{0}>1$, time delay accelerates the spreading of disease. Our theoretical studies deliver some new insights for understanding the propagation of diseases and information in biological and social networks.

## Supporting Information

S1 FigThe BA network $G_{1}$ with 800 vertexes.(PDF)

S2 Fig The degree distribution of the network. (PDF)

S3 FigThe dynamical behaviors of the susceptible nodes when $R_{0}=0.7025$.(PDF)

S4 FigThe dynamical behaviors of the infected nodes when $R_{0}=0.7025$.(PDF)

S5 FigThe dynamical behaviors of the susceptible nodes when $R_{0}=1.5680$.(PDF)

S6 FigThe dynamical behaviors of the infected nodes when $R_{0}=1.5680$.(PDF)

S7 FigThe trajectories of *I*(*t*) of systems Eqs (37), (39) and (40) when $R_{0}\leq 1$.(PDF)

S8 FigThe trajectories of *I*(*t*) of systems Eqs (41), (42) and (43) when $R_{0}>1$.(PDF)
