# Supplementary figures and images for: Stability Analysis of SIR Model with Distributed Delay on Complex Networks
Source: PLoS One. 2016 Aug 4;11(8):e0158813. doi: 10.1371/journal.pone.0158813 (PMC4973911; doi:10.1371/journal.pone.0158813)

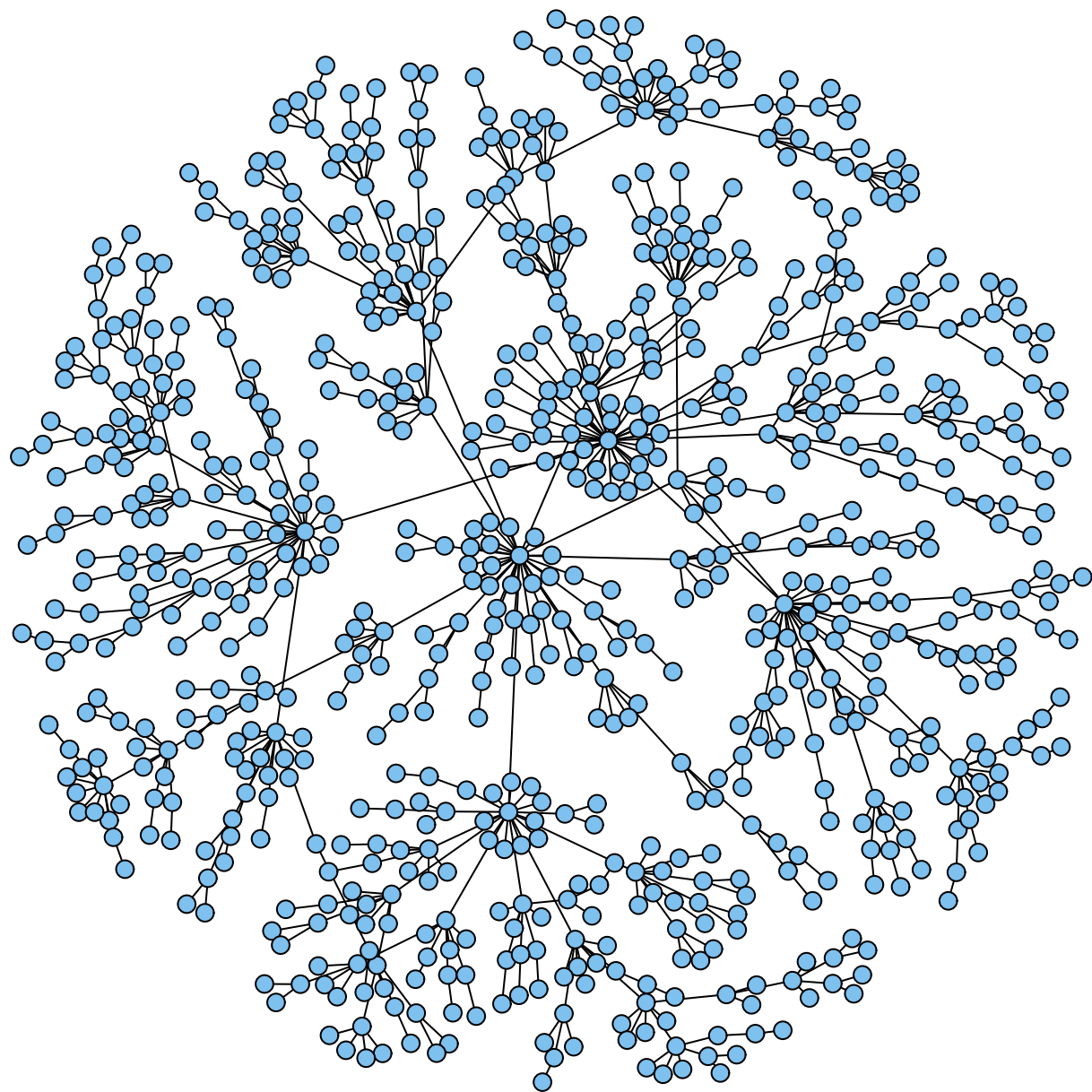

Supplement: S1 Fig — (PDF) [file pone.0158813.s001.pdf]

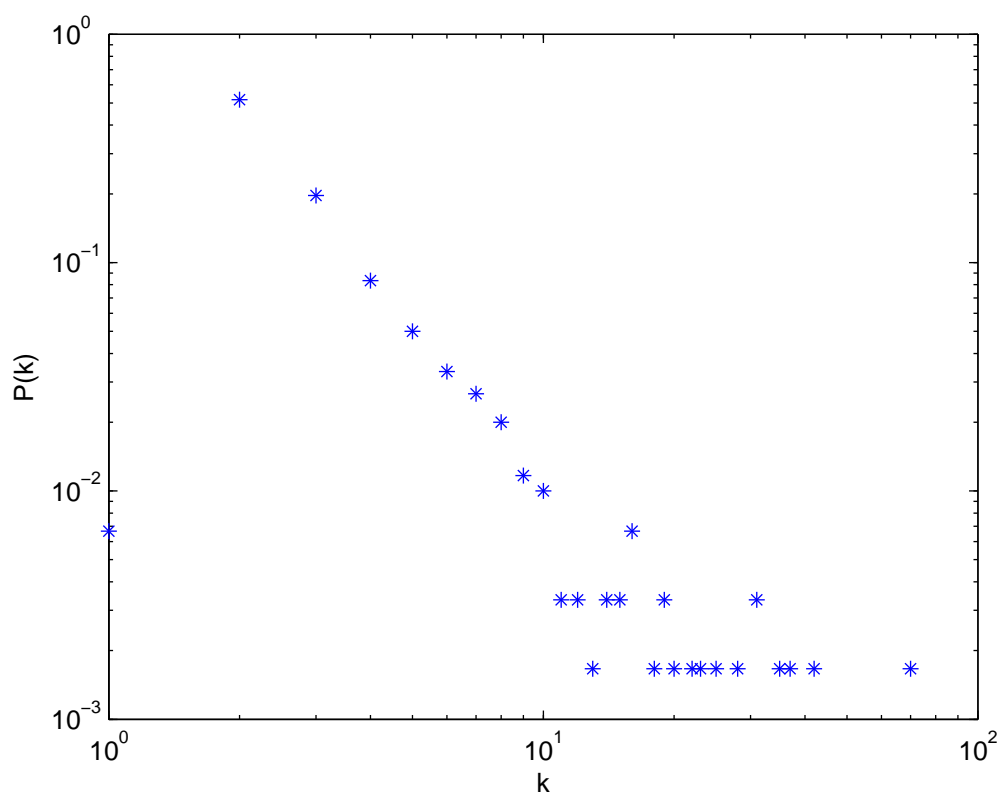

Supplement: S2 Fig — (PDF) [file pone.0158813.s002.pdf]

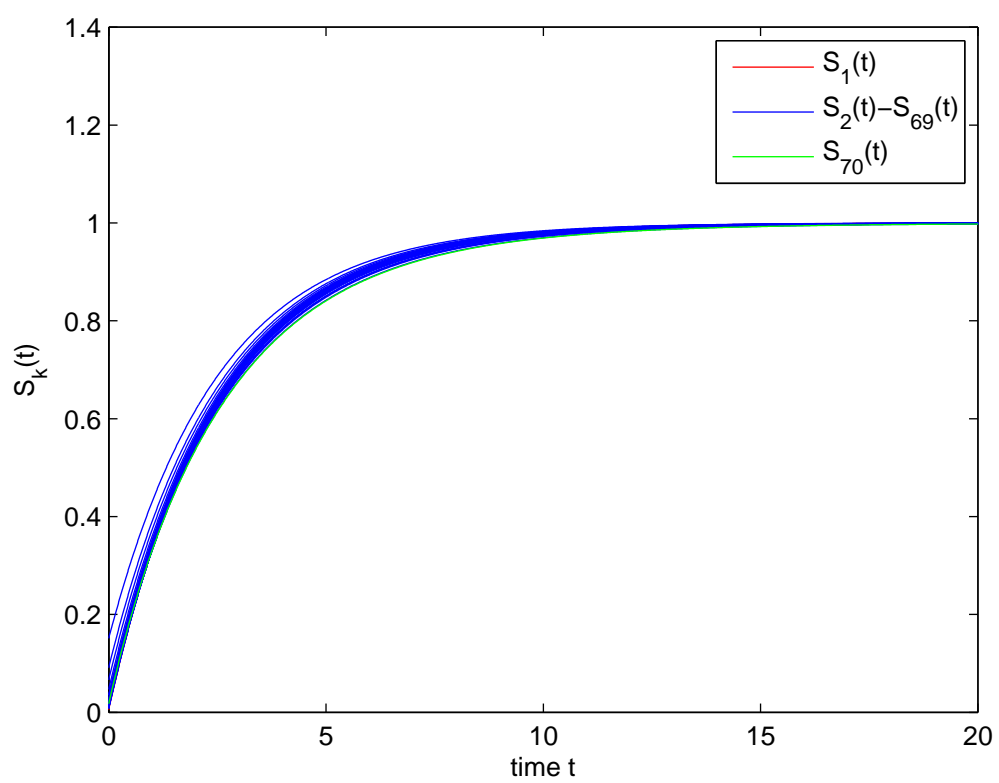

Supplement: S3 Fig — (PDF) [file pone.0158813.s003.pdf]

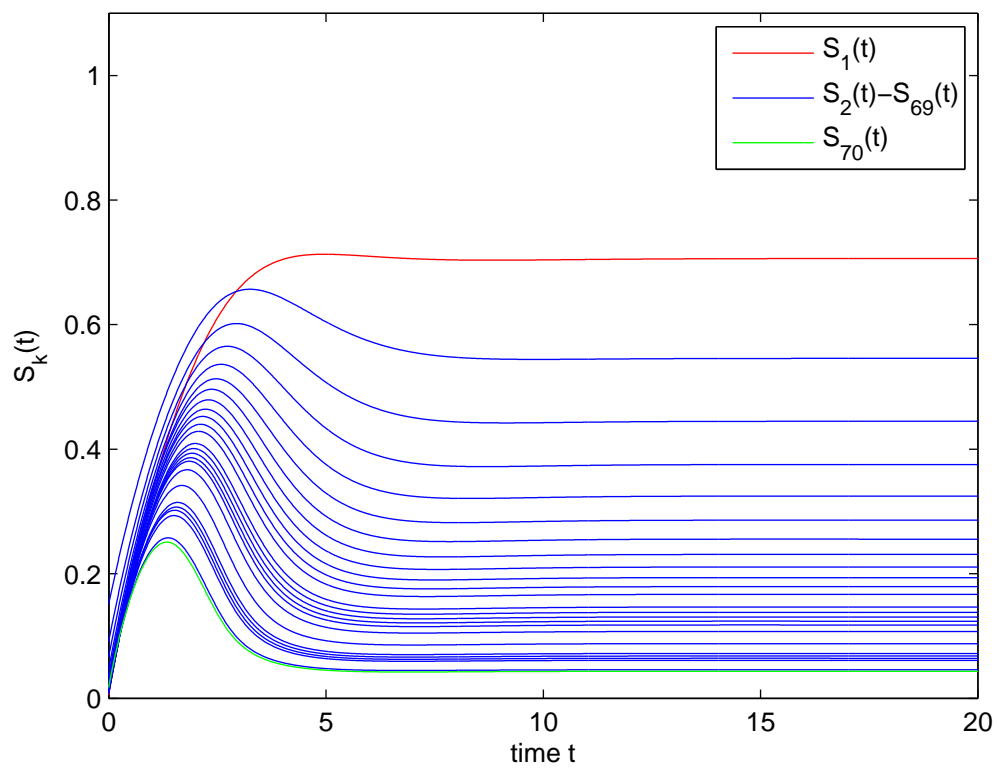

Supplement: S4 Fig — (PDF) [file pone.0158813.s004.pdf]

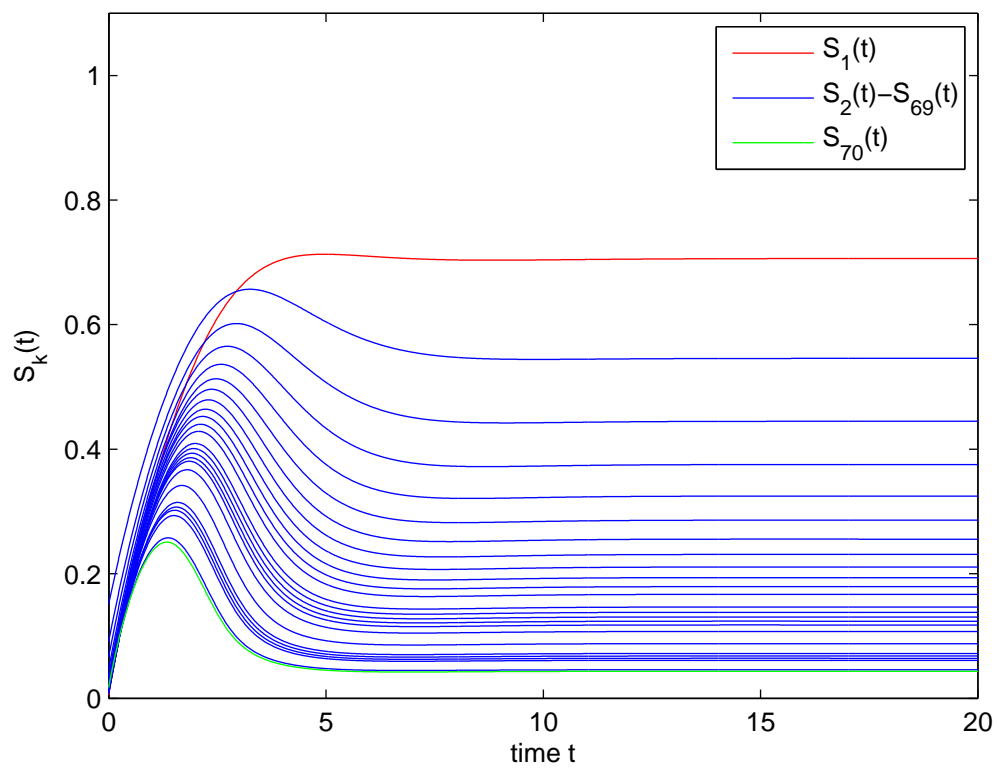

Supplement: S5 Fig — (PDF) [file pone.0158813.s005.pdf]

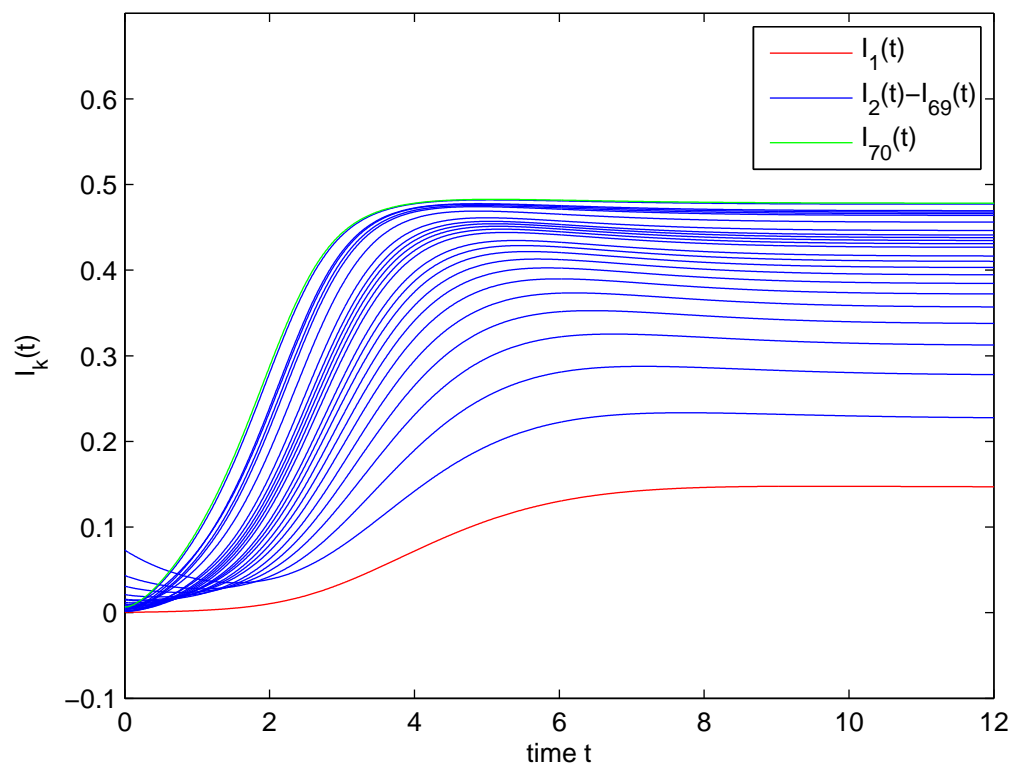

Supplement: S6 Fig — (PDF) [file pone.0158813.s006.pdf]

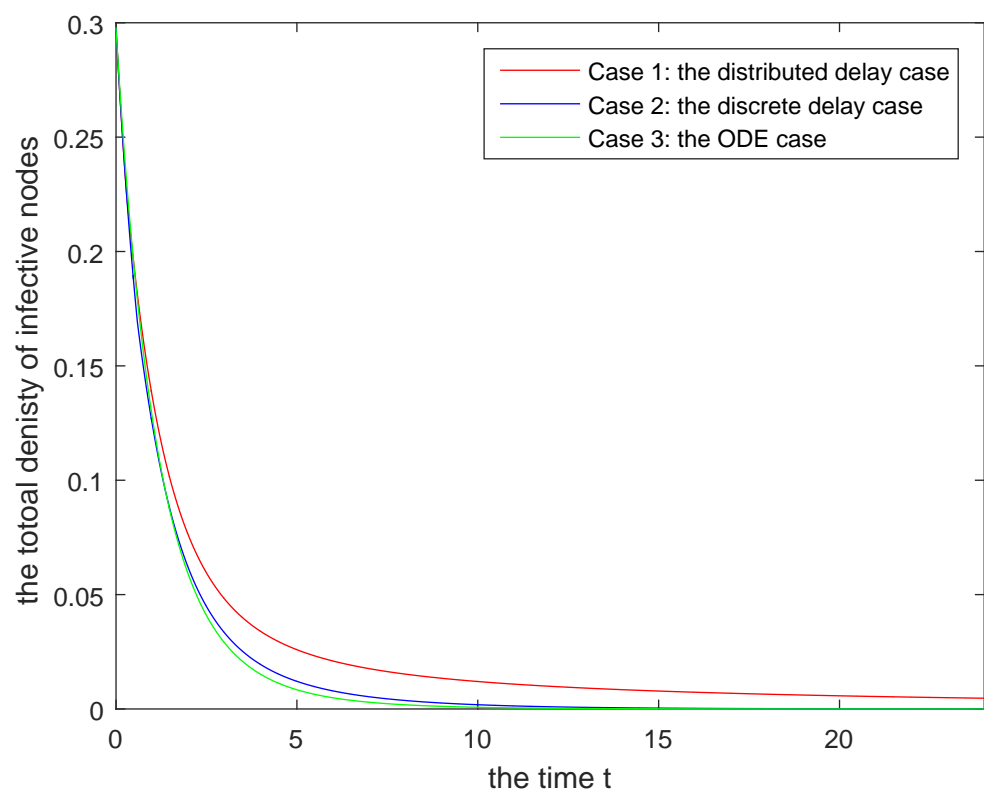

Supplement: S7 Fig — (PDF) [file pone.0158813.s007.pdf]

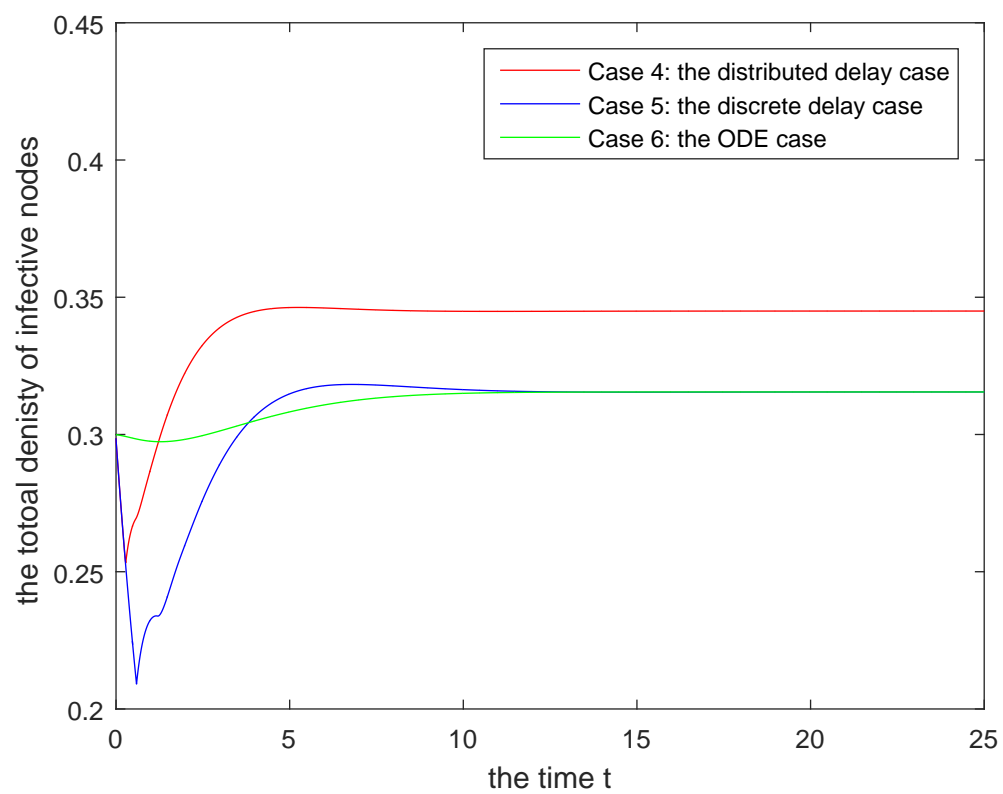

Supplement: S8 Fig — (PDF) [file pone.0158813.s008.pdf]
